# Stagnation leads to short-term fluctuations in the effluent water quality of biofilters: A problem for greywater reuse?

**DOI:** 10.1016/j.wroa.2021.100120

**Published:** 2021-09-15

**Authors:** Angelika Hess, Chiara Baum, Konstanze Schiessl, Michael D. Besmer, Frederik Hammes, Eberhard Morgenroth

**Affiliations:** a Eawag: Swiss Federal Institute of Aquatic Science and Technology, 8600 Dübendorf, Switzerland; b ETH Zürich, Institute of Environmental Engineering, 8093 Zürich, Switzerland; c onCyt Microbiology AG, 8038 Zürich, Switzerland

**Keywords:** Biological activated carbon, Greywater reuse, Automated flow cytometry, Detachment, Stagnation

## Abstract

A key characteristic of decentralized greywater treatment and reuse is high variability in both nutrient concentrations and flow. This variability in flow leads to stagnant water in the system and causes short-term fluctuations in the effluent water quality. Automated monitoring tools provide data to understand the mechanisms underlying the dynamics and to adapt control strategies accordingly. We investigated the fluctuations in a building-scale greywater treatment system comprising a membrane bioreactor followed by a biological activated carbon filter. Short-term dynamics in the effluent of the biological activated carbon filter were monitored with automated flow cytometry and turbidity, and the impact of these fluctuations on various hygiene-relevant parameters in the reuse water was evaluated. Continuous biofilm detachment into the stagnant water in the biological activated carbon filter led to temporarily increased turbidity and cell concentrations in the effluent after periods of stagnation. The fluctuations in cell concentrations were consistent with a model assuming higher detachment rates during flow than during times with stagnant water. For this system, total cell concentration and turbidity were strongly correlated. We also showed that the observed increase in cell concentration was not related to either an increase of organic carbon concentration or the concentration of two opportunistic pathogens, *P. aeruginosa* and *L. pneumophila.* Our findings demonstrate that turbidity measurements are sensitive to changes in the effluent water quality and can be used to monitor the fluctuations caused by intermittent flow. Intermittent flow did not lead to an increase in opportunistic pathogens, and this study provides no indications that stagnant water in biological activated carbon filters need be prevented.

## Highlights

• A greywater treatment system was monitored with automated flow cytometry and turbidity.
• Stagnation in biological activated carbon filter led to peaks in total cell concentration and turbidity.
• Strong correlation was observed between TCC and turbidity.
• Stagnation did not lead to increase of opportunistic pathogens in the biofilter's effluent.

## Graphical abstract

**Figure:**
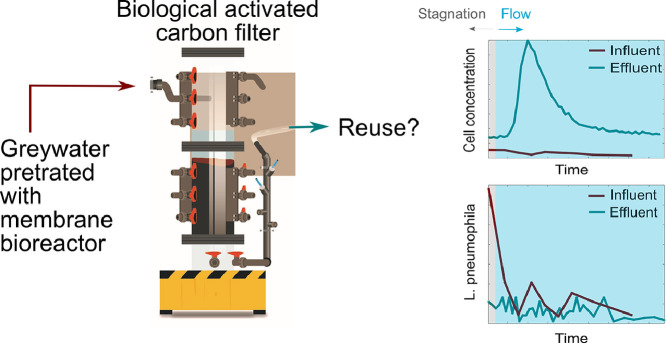

## 1 Introduction

Greywater reuse for non-potable applications such as washing machines and showers must provide hygienically acceptable water at all times. This requirement renders the influence of short-term fluctuations in treated water quality important. One promising technology for buffering influent variability in biodegradable and adsorbable compounds is the biological activated carbon (BAC) filter. Highly variable flow and stagnation have been shown to have no negative impact on total organic carbon (TOC) removal (Hess et al. 2020, McKie et al. 2019) and BAC filters can be implemented to improve the biological stability of the water (Ziemba et al. 2020). However, the biological degradation of organic carbon is linked to bacterial growth and biofilm formation in the BAC (Velten et al. 2011) where biofilm growth and detachment can in turn lead to fluctuations in the effluent water quality.

Typically, greywater is treated in a decentralized way, ranging from single-household and building scale up to neighborhood scale. The smaller the scale, the higher will be the variability in both the flow and composition of the influent into the greywater treatment system (Gross 2015). A time lag between the production and reuse of greywater and irregular usage patterns inevitably lead to stagnant water in treatment systems, in pipes, and in clean water storage tanks. An effective treatment step in retaining bacterial pathogens is a membrane bioreactor (MBR) (Wu 2019). Nevertheless, opportunistic pathogens such as *P. aeruginosa* and *L. pneumophila* can grow after treatment due to remaining nutrients and nonsterile conditions and can cause serious hygienic problems for greywater reuse (Blanky et al. 2017, Friedler et al. 2011). Recurring stagnation can increase cell concentrations due to microbial detachment during and after stagnation, for example in intermittent drinking water supply systems (Bautista-de los Santos et al. 2019, Kumpel and Nelson 2016) and in building plumbing (Bédard et al. 2018, Lautenschlager et al. 2010, Lipphaus et al. 2014). However, the extent to which intermittent flow and biofilm detachment in the biofilter lead to a deterioration of hygiene-relevant parameters such as the concentration of opportunistic pathogens remains unclear.

Biofilm detaches into bulk water both as single cells and through sloughing off of biofilm caused by shear stress (Davies 2011). Shear stress can detach biofilm that formed in stagnant water when flow starts again (Bédard et al. 2018). In tap water, for example, cell concentration is elevated after periods of stagnant water, and the frequency with which flow is interrupted affects the extent to which bacterial numbers increase (Lipphaus et al. 2014). This may be explained by the fact that detachment is influenced by the hydrodynamic conditions under which biofilm forms (Abe et al. 2012) and the biofilm adapting to changing shear conditions (Choi and Morgenroth 2003). Cell concentrations can be increased after stagnation by changes in shear stress and by continuous detachment, both leading to increased bulk concentrations that are washed out with the first flush (Besmer and Hammes 2016). Therefore, it is important to quantify and understand these cell concentrations in greywater treatment systems that operate with intermittent flow.

Automated online flow cytometry (FCM) is a valuable tool for investigating and monitoring short-term microbial fluctuations such as increased cell concentrations after detachment (Besmer et al. 2014). The high temporal resolution allows a better understanding of the underlying microbial processes in for example drinking water treatment (Besmer and Hammes 2016, Buysschaert et al. 2018) and stagnant water in the distribution network (Farhat et al. 2020). Even though greywater treatment systems have to deal with high variability, to our knowledge, no online microbial measurements have been applied so far to study microbial water quality in greywater treatment systems. Whereas online FCM requires specialized sophisticated equipment and is not currently widespread, online turbidity measurements are rather simple and routinely applied to monitor biofilters (Hooper et al. 2019). Turbidity and FCM both measure light scattering caused by suspended particles. FCM additionally measures fluorescence. Fluorescent stains can be used to distinguish bacterial cells from other particles (Safford and Bischel 2019). Turbidity sensors are unable to distinguish between microbial and nonmicrobial particles. Depending on the system monitored, turbidity may be a suitable indicator of microbial water quality or not. Page et al. (2017) showed that turbidity and total cell concentration (TCC) followed similar dynamics in a karst spring. In contrast, Ikonen et al. (2013), McCoy and Olson (1986), Prest et al. (2021) showed only a weak correlation between microbial water quality and turbidity for a drinking water distribution system and a municipal drinking water treatment plant. Combining online FCM with turbidity measurements provides a unique opportunity to compare turbidity and total cell concentration in a real-life system with intermittent flow and highly variable nutrient concentrations.

The objective of this study was to better understand how stagnation, an inherent consequence of the highly variable flow in greywater treatment systems, influences the effluent water quality of a BAC filter. We measured cell concentrations in the effluent of a greywater treatment system over a two-week period at high temporal resolution (5 min) to quantify short-term fluctuations. We used these data to evaluate the extent to which turbidity was related to cell concentration. We hypothesized that high cell concentration after stagnation is caused by continuous detachment of cells from the BAC filter. Furthermore, we assessed the extent to which the dynamics of cell concentrations are reflected in the dynamics of two opportunistic pathogens before and after the BAC filter.

## 2 Material and methods

### 2.1 Experimental setup

**Fig. 1:**
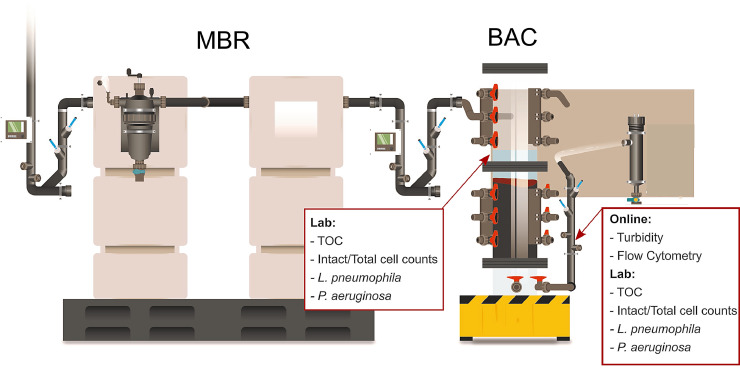
Greywater treatment system in the Water Hub at NEST. A membrane bioreactor (MBR) is followed by a biological activated carbon (BAC) filter for post-treatment. For this study, measurements were taken at the top of the BAC and in the BAC effluent pipe (Illustration: Peter Penicka, Eawag).

The Water Hub at NEST is a platform for investigating technologies for source-separated wastewater streams in buildings (grey-, yellow-, and blackwater) (Etter et al. 2016). On average, 342 L/d of greywater produced in the NEST building is treated in two steps: a membrane bioreactor (MBR) with an ultrafiltration membrane is followed by a biological activated carbon (BAC) filter (Figure 1). The greywater originated from a fitness unit, flats, and public bathrooms, and its composition was highly variable (Table 1).

**Table 1:** Water quality across the treatment system. Values are medians with 25 and 75 percentiles in parentheses. The data over time can be found in Supporting Information S 1.2. TOC, NH_4_-N, NO_3_-N, and PO_4_-P were measured in the laboratory, n is the number of analyzed samples. pH was measured online with a time resolution of 4–5 s.

	TOC (mg/L)	NH_4_-N (mg/L)	NO_3_-N (mg/L)	PO_4_-P (mg/L)	pH (-)
Raw greywater (n = 70)	24.7 (16, 38)	1.3 (0.5, 5.5)	2.3 (1.3, 3.2)	0.2 (0.2, 0.5)	7.7 (7.5, 8.0)
After MBR (n = 197)	3.8 (3.0, 5.0)	0.015 (0.015, 0.27)	10.2 (6.5, 14.1)	1.5 (1.0, 2.5)	8.1 (7.8, 8.2)
After BAC (n = 239)	1.0 (0.63, 1.4)	0.015 (0.015, 0.06)	10.0 (6.9, 14.1)	1.5 (0.9, 2.2)	7.6 (7.5, 7.7)

The MBR is a modified version of a commercially available system (Aquacell 800, newterra). The MBR consists of two tanks with mean hydraulic residence times of 13 h in the first tank and 17 h in the second. The highly variable influent flow rate and the on-off operation of the MBR lead to intermittent flow into the BAC filter. The BAC filter (column material: PVC, granular activated carbon (GAC): Chemviron F-400) is operated with a mean empty bed contact time (EBCT) of 112 min and a mean hydraulic loading rate of 0.4 m/h. The system has been monitored with both laboratory and online measurements for a period of more than two years (Table 1). The BAC filter was operated for 550 days before the measurement campaign with online FCM was started, corresponding to 5900 treated empty bed volumes. The MBR retains pathogens: we obtained more than 5.5 log10 removal of *Enterococcus* with the MBR. Further, the MBR degrades organic carbon and nitrifies ammonium to nitrate. The BAC removes the residual organic carbon and is operated with gravity-driven down-flow and without backwashing.

For this study, BAC influent samples were taken from the water at the top of the filter bed and effluent samples from the effluent pipe (material: PE-HD) as indicated in Figure 1.

### 2.2 Turbidity

Turbidity was measured with a Turbimax CUS52D (Endress + Hauser); this is a sensor for drinking and process waters with a detection limit of 0.0015 FNU (Formazin Nephelometric Unit) and therefore suitable for low turbidity measurements. The sensor was installed directly in the up-flow pipe (D = 63 mm) after the BAC (photo in Supporting Information S1.1). The sensor measures a data point every 5 s. All data points <= 0 FNU and >=2 FNU or differing more than 0.03 FNU from the moving average over 15 data points were considered to be outliers. Some 3% of the data points were labelled as outliers and were excluded from the data set.

### 2.3 Automated online flow cytometry

#### 2.3.1 Measurements

Over a period of two weeks, the effluent of the BAC was analyzed every 5 minutes for total cell concentration (TCC). TCC was measured with a BD Accuri C6 Plus flow cytometer and the automation unit onCyt DOFCM-200 (onCyt, Switzerland) as described previously (Besmer et al. 2014). The samples were mixed with a fluorescent nucleic acid stain (SYBR Green I; final concentration 1:10,000 in TRIS buffer containing 50 mM sodium thiosulfate). The mixture was then incubated for 10 min at 37°C and subsequently transferred to the flow cytometer for a measurement of 99 µl at a flow rate of 66 µL/min. After each sampling and measurement, the staining module and the flow cytometer were rinsed with ultrapure water.

#### 2.3.2 Data analysis

Data was analyzed with the customized software cyPlot (onCyt, Switzerland). The density plots were visually inspected to exclude abnormal density plots from the data set. A more detailed description of the abnormal density plots can be found in Supporting Information S 1.3. In total, from 3995 data points, 140 data points (4 %) were identified as outliers and removed from the data set.

### 2.4 Laboratory measurements

Laboratory measurements were taken to characterize one of the peak events in more detail. The effluent of the BAC was sampled first every 5 minutes and afterwards every 15 minutes. An influent sample was taken for every fourth effluent sample.

#### 2.4.1 Flow cytometry

Samples for conventional flow cytometry were taken in autoclaved glassware. Samples were fixed with glutaraldehyde (final concentration: 0.1 %) and formaldehyde (final concentration: 0.01 %) and kept in the dark at 4°C until analysis. TCC and intact cell concentration (ICC) were measured with a CytoFLEX (Beckman Coulter, Brea, California, USA) at a flow rate of 60 µL /min for 30 s. TCC was measured with SYBR Green I stain (final concentration 1:10,000 in TRIS buffer), and for ICC, propidium iodide (PI) was added (final concentration: 6 µM). All samples were measured in triplicate. The TCC measured with conventional flow cytometry were also compared to TCC measured with online flow cytometry (Supporting Information S 1.4). Both show the same trends but differ somewhat in the absolute numbers measured.

#### 2.4.2 Legionella pneumophila

Legiolert (IDEXX Laboratories, USA) was used to quantify the most probable number (MPN) of *Legionella pneumophila* in the water samples following the “nonpotable water” protocol provided by the manufacturer. Samples were processed within 4 h after sampling.

#### 2.4.3 Pseudomonas aeruginosa

*P. aeruginosa* was detected with GSP (Sigma-Aldrich) selective agar with addition of penicillin G (Fluka), i.e., benzylpenicillin sodium. A 1:1000 dilution with nanopure water was used for filtration, and the membrane filters on the agar plates were incubated at 28°C for 24–29.5 h. After incubation, the number of colony-forming units (CFUs) that had grown on the filter were counted. No further confirmatory assays were performed. All glassware used was autoclaved. Samples were processed within 4 h after sampling, and all samples were taken in duplicate.

#### 2.4.4 Total organic carbon

TOC was measured using a total organic carbon analyzer (Shimadzu TOC-L, Kyoto, Japan) within 24 h after sampling and the samples were kept at 4°C until analysis. All glassware used was muffled at 450°C for 4 h.

### 2.5 Model

Model calculations were performed to test whether hypothesized continuous bacterial detachment in the BAC filter is compatible with observations. We hypothesized that continuous bacterial detachment during periods of stagnation leads to an accumulation of cells in the bulk water of the BAC filter, and that these are washed out when flow starts.

#### 2.5.1 Hydraulic

The BAC filter was modelled as a series of ideal reactors. The water volume on top of the filter bed was modelled as a completely mixed tank with variable volume. The BAC filter was modelled as a series of completely stirred tank reactors (CSTRs) to represent a plug flow reactor (PFR) with dispersion. The volume at the bottom of the filter bed was modelled as a single CSTR, and that of the pipe to the measurement and sampling point was modelled as a PFR. The flow through the BAC (Q_out_) was calculated from the pressure changes measured on top of the filter bed. The details for the calculations can be found in Supporting Information S 1.5. The only fitted parameter, number of CSTRs to represent PFR, was determined from visual comparison of the hydraulic model and a tracer test using NaCl (Supporting Information S 1.5.3).

#### 2.5.2 Detachment

In this study, we modelled the detachment of cells as a function of biomass concentration. Growth and decay of cells in the BAC filter were not modelled explicitly. The detachment was calculated with a detachment constant and relative to the adenosine triphosphate (ATP) concentration on the GAC (Supporting Information S 1.5.4), which changes over the filter bed:

$$\Delta c_{D}=k_{D}\cdot ATP_{n}\cdot \Delta t$$

where $\Delta c_{D}$ = change of cell concentration in the bulk phase caused by detachment (#/mL); $k_{D}$ = detachment coefficient (# g_GAC_ mL^−1^ g_ATP_^−1^ s^−1^); $ATP_{n}$ = ATP concentration on the GAC in the n^th^ compartment of the filter (g_ATP_ g_GAC_^−1^); Δt = time step for the model calculations (s).

Two modelling approaches are discussed in this publication, one with a constant k_D_ and the other with a higher k_D_ during times with flow and a lower k_D_ during stagnation. The coefficients were determined by visual comparison of the measured and the calculated TCC concentrations. The focus was on i) the peak height and ii) the baseline TCC values. The coefficients determined for the models are listed in Table 2.

**Table 2:** Table 2

	k_D_ (# g_GAC_ mL^−1^ g_ATP_^−1^s^−1^)	
	Q_out_ = 0	Q_out_ > 0
Model with constant k_D_	4.0⋅10^5^	4.0⋅10^5^
Model with variable k_D_	1.8⋅10^5^	4.5⋅10^5^

## 3 Results

### 3.1 Stagnation leads to increased turbidity and TCC

**Fig. 2:**
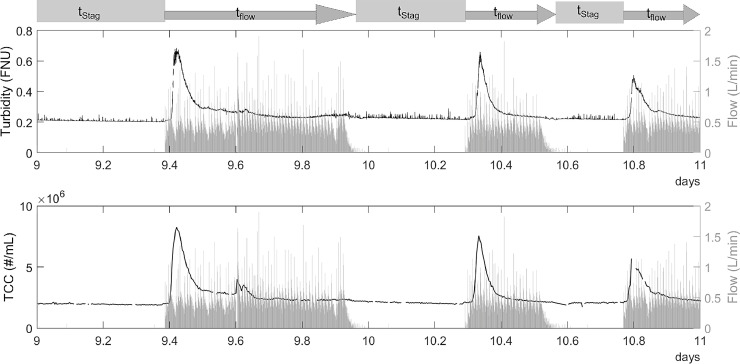
Turbidity, TCC (both in black), and flow (grey) in the effluent of the BAC shown for a period of two days. Above the plots, the stagnation time (t_Stag_) and the time with flow are shown, also indicating the time since stagnation (t_flow_). Clear peaks are visible in total cell concentration and turbidity after stagnation periods.

Highly variable inflow to the greywater system and the operation of the MBR led to intermittent flow to the BAC. This intermittent flow led to periods with stagnant water in the BAC filter. When flow resumed after stagnation, both turbidity and total cell concentration (TCC) showed increased values in the BAC effluent (Figure 2). The effluent concentrations in TCC and turbidity then gradually decreased to the values measured before stagnation. These data show that after stagnation, cells were washed out from the BAC. TCC was constant during periods with stagnant water, indicating no growth in the stagnant water in the pipe after the BAC.

**Fig. 3:**
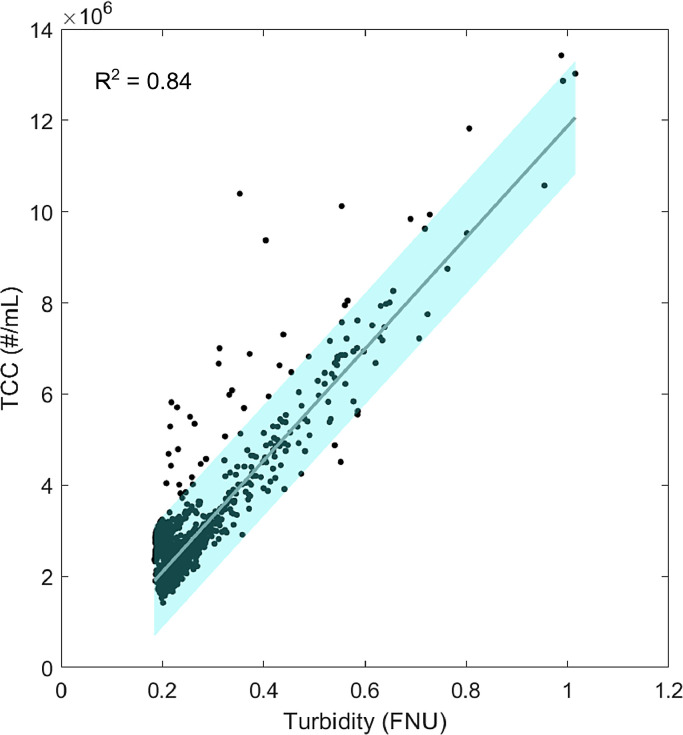
Correlation between turbidity and total cell concentration over two weeks measured (n = 3960). The 95% confidence prediction interval is shown in light blue.

TCC and turbidity followed the same pattern with peaks after stagnation. Data from two weeks of online measurements show strong, statistically significant (p<0.05) correlation (R^2^ = 0.84) between TCC and turbidity (Figure 3). Nonetheless, a single turbidity value cannot indicate the exact cell concentration.

### 3.2 Longer stagnation periods are linked to higher TCC in the effluent

**Fig. 4:**
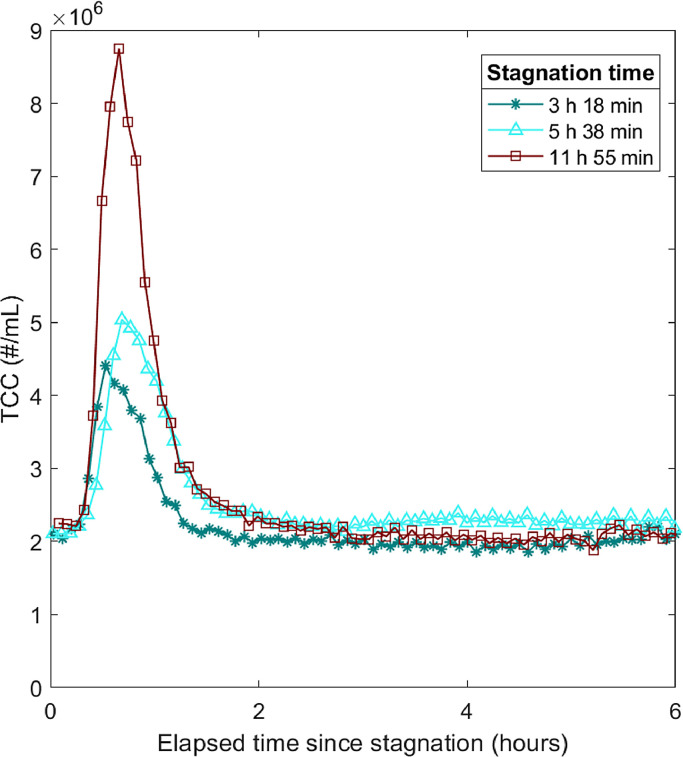
Influence of stagnation time on the effluent values for cell concentration. Here, three TCC peaks are shown out of the 14 peaks measured for three stagnation times. The time with flow before the stagnation was around 12 h for all three peaks.

The longer the water stagnated prior to flow, the higher were the measured TCC and turbidity after the flow through the filter started again. As an example, Figure 4 depicts how for TCC both the peak height and the area below the peak were higher for longer stagnation periods. Therefore, both the maximum concentration of bacteria and the total bacterial load were increased after stagnation.

**Fig. 5:**
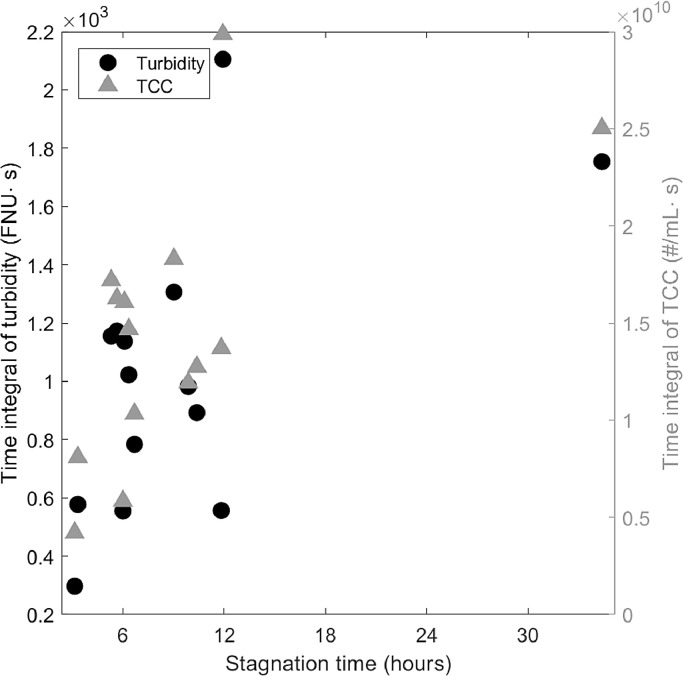
Area below the curves of turbidity and TCC (total bacterial load) for a range of stagnation times.

Figure 5 shows the area below the different peak events for turbidity and TCC for different stagnation times for all the peaks that occurred over a period of 15 days. The area for each peak event is calculated as the integral between the peak curve and a baseline. The baseline is defined as the mean value during the first 8 minutes after flow starts again. The data clearly show that the area below the curve for both turbidity and TCC increased with stagnation duration. The coefficients of determination (R^2^) are 0. 56 (*p* = 0.04) for turbidity and 0.59 (*p* = 0.02) for TCC.

### 3.3 Continuous detachment of cells from the BAC

**Fig. 6:**
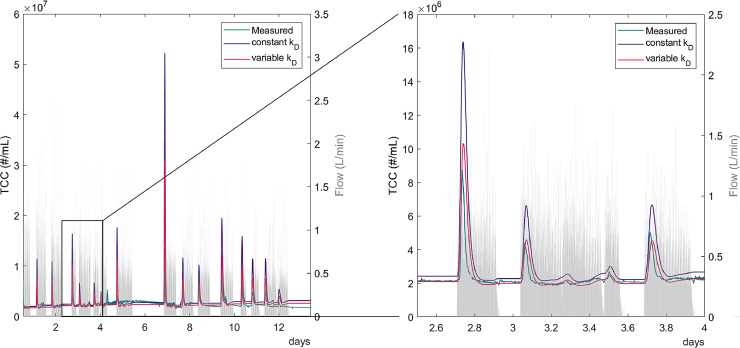
Comparison of measured and modelled cell concentrations in the effluent of the BAC. One model is with constant detachment (k_D_) and the other with variable detachment (k_D_ 2.5 times higher during flow than during stagnation) in the BAC filter. The right plot shows a magnification over 1.5 days.

Simple model calculations were used to test the assumption of continuous cell detachment from the BAC. The measured cell concentration was compared with two model configurations, both assuming that detachment always occurs: i) constant detachment independent of the flow, and ii) higher detachment during flow than during stagnation. The model with higher detachment during times with flow than during periods of stagnation is able to represent the general patterns of measured cell concentrations (Figure 6).

Both the constant- and varying-detachment models present peaks with a slight delay compared to the measured data (Supporting Information S 2.3). The model with constant detachment overestimates the maximum peak heights, whereas that with two detachment rates is more accurate. Both models indicate that even a short decrease in flow leads to increased cell concentration in the effluent afterwards. The delay between start of flow and the peak maximum was caused by hydraulics: the sampling and measuring location is not directly below the filter bed. The model shows that during stagnation the TCC in the filter bed also increased due to continuous detachment during times of stagnation. These cells were then washed out of the filter when flow started again.

### 3.4 Relevance for hygiene of the reuse water

**Fig. 7:**
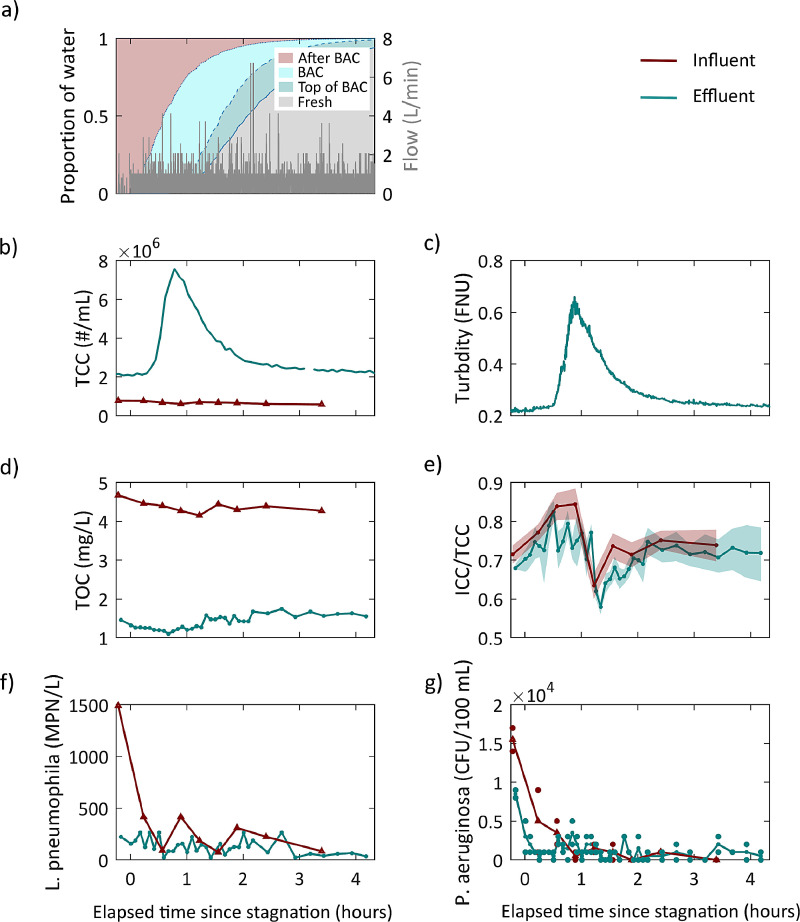
Parameters sampled during one turbidity peak in the influent and effluent of the BAC. a) The proportion of water that stagnated in different parts of the system and flow. b) TCC in influent and effluent. c) Turbidity during the sampling. d) Organic carbon in the influent and effluent. e) The ratio of ICC to TCC, measured in triplicate; the error band shows the standard deviation. f) and g) Presumptive positive concentration of opportunistic pathogens.

Detachment of cells from the BAC filter can also be detected in data from turbidity measurements. To assess the water quality during the increased turbidity for hygiene-relevant parameters, one peak-event was sampled for TCC, ICC, TOC, *L. pneumophila,* and *P. aeruginosa* (Figure 7).

The washout of water that had stagnated in different locations of the system is shown over time in Figure 7a. The hydraulic model that was used to calculate washout is presented in Supporting Information S 1.5. When the flow started, water that had stagnated after the BAC was sampled first. Over time, the proportion of water that had stagnated in the filter bed increased in the flow. This proportion reached its maximum at the same time that the peak values for TCC and turbidity were reached. When a higher proportion of the water that either stagnated on top of the filter bed or was fresh MBR permeate reached the sampling location, TCC decreased again to previously observed values. We therefore concluded that the water that stagnated in the filter bed had the highest TCC and turbidity.

The influent concentration for TCC was constant during the whole sampling period, showing that the changes in the effluent were caused by processes in the filter itself and not processes in the pipes before, as also observed by Zhang et al. (2015). The effluent TCC was higher than the influent concentration over the whole period, indicating microbial growth in the BAC filter.

The turbidity during the sampled peak event showed the typical pattern as observed for previous peak curves, indicating that the more frequent sampling did not influence the observed effluent water quality significantly. The influent turbidity was not measured on the day of more frequent sampling, but previous measurements had shown that the influent turbidity had an mean of 0.05 (SD = 0.02) FNU and is thus lower than the effluent turbidity at on mean 0.23 FNU (SD = 0.06).

The ratio between intact and total cell concentration did not change significantly between influent and effluent. Both influent and effluent showed a decrease in the fraction of intact cells around 1.5 h after flow onset. The lowest ratio occurred after the peak in TCC was measured. Less than 1 h after the turbidity peak, the ratio increased again to values similar to those observed at the beginning of the sampling.

Throughout the whole measurement period, a significant removal (on average 68%) of organic carbon was observed. During the peak event of TCC, no increase in TOC in the effluent was measured. In contrast, the TOC effluent concentrations were slightly lower during this period. The TOC in the influent did not change significantly over the 4 h of sampling.

The curves measured for the opportunistic pathogens, *L. pneumophila* and *P. aeruginosa*, look comparable. No increase in presumptive positive concentrations was observed for either from the influent to the effluent of the BAC filter. Also, no increase was observed with the increased TCC. The highest concentrations, 1490 MPN/L and 15500 CFU/100mL, for presumptive positive *L. pneumophila* and *P. aeruginosa* respectively, were measured in the water that stagnated on top of the filter bed. *P. aeruginosa* also showed a high cell concentration in the sample that stagnated in the effluent pipe prior to sampling, whereas this was not the case for *L. pneumophila*.

Overall, only turbidity and ICC correlated significantly with TCC measured in the effluent. Further, there was a negative correlation between TOC and ICC (R = -0.39). The correlation matrix is given in Supporting Information S 2.4.

## 4 Discussion

### 4.1 Stagnation leads to increased turbidity and TCC

In biofilters, organic carbon is degraded, and thus microbial growth is to be expected. Not all these cells accumulate in the filter, and instead most of the bacteria are washed out of the filter, leading to higher cell concentrations in the effluent than in the influent of the BAC (Velten et al. 2011). A sudden increase in flow velocity and thus shear stress or intermittent flow conditions have been shown to lead to increased bacterial concentration in the effluent of GACs (Besmer and Hammes 2016, Zhang et al. 2015). Therefore, we were not surprised to observe increased cell concentration after periods with stagnation in the effluent of our treatment system. Hence, even though the MBR degrades a substantial portion of the organic carbon, enough nutrients remain to support subsequent growth afterwards. Therefore, the questions are not whether growth occurs but where the remaining carbon is degraded; whether most of the bacteria grow in biofilter, pipes, or storage tanks; and whether the growth is problematic from a water quality perspective, for example due to increased pathogen concentrations. A biofilter allows more spatially concentrated growth, which provides more opportunities for controlling and monitoring growth and detachment. Such monitoring might, for instance, be used to initiate the recirculation of water of insufficient quality.

Data from the BAC filter effluent showed a strong correlation between turbidity and total cell concentration. This is somewhat unusual, because most systems incorporate a wide variety of turbidity-causing materials (Farrell et al. 2018). Here, the ultrafiltration membrane's pore size of 0.04 µm retains most materials captured by turbidity measurements, including bacteria. All the cells and other turbidity-causing material can therefore be assumed to originate from the pipes between the MBR and the BAC and from the BAC filter itself. An SEM microscopy analysis of the water at peak turbidity showed very few particles other than cells. A few GAC, silicate, and chalk particles were observed in the BAC effluent (Supporting Information S 2.1). However, the number of particles was too low to explain the increase in turbidity. Moreover, from an experiment with a dilution series of a bacterial pure culture, we confirmed that turbidity correlated well with total cell concentration at the concentrations we observed here (data shown in Supporting Information S 2.2). These additional measurements can explain the strong correlation of turbidity with TCC after the BAC. We therefore particularly recommend the use of turbidity sensors in treatment systems with membranes as an easy way to monitor growth and detachment in a biofilter. Concentrating growth and therefore detachment spatially in a biofilter allows a turbidity sensor to monitor these processes. An unexpected change in turbidity caused, for example, by growth during stagnation or breakthrough of bacteria through the filter can be used as a warning signal for process failure and should be followed by a closer investigation on whether the water is still safe for reuse. Which type of sensors can best detect changes in water quality and process failures depends on the system that is monitored.

### 4.2 Longer stagnation periods are linked to higher TCC in the effluent

Typically, engineered biofilters are designed to be operated without water stagnating in the filter. Therefore, little research has been published about the effects of stagnation on the effluent water quality of BACs. These publications have focused on the overall removal performance of organic carbon and how stagnation influences the biological activity and the sorption capacity on the filter but have not studied short-term fluctuations (Hess et al. 2020, McKie et al. 2019). Other systems with regular change between flow and stagnation include building plumbing (Lautenschlager et al. 2010) and drinking water systems with intermittent supply (Bautista-de los Santos et al. 2019). Studies in building plumbing have shown that the longer the stagnation and the more biofilm in the pipe, the higher is the cell concentration in the effluent after stagnation (Lautenschlager et al. 2010, Proctor 2017). A deterioration in water quality is also to be expected in intermittent water supply after water stagnates, and sometimes pipes even run dry due to interrupted water supply (Kumpel and Nelson 2016). Therefore, we were not surprised to observe increased TCC and turbidity in the effluent of a biofilter. Nevertheless, there are also important differences. In pipe systems, cell concentration did not further increase for stagnation times longer than 12 hours (Lautenschlager et al. 2010), but in the BAC, peak cell concentration continued to increase with stagnation time. Despite the similarities in the systems, differences between pipes and biofilters could also influence the detachment of bacteria, such as the surface-to-volume ratio (Bédard et al. 2018) and the flow conditions (Picioreanu et al. 2001).

### 4.3 Continuous detachment of cells from the BAC

The fact that higher cell concentrations were measured in the effluent after longer stagnation indicates that cells detach continuously even during periods without flow. A model with continuous detachment of cells in the BAC and higher detachment during periods with flow than during stagnation can explain these peak events. The results of this study suggest that a combination of continuous detachment of cells from the biofilm and increased detachment caused by shear stress during times with flow lead to peaks in cell concentrations after stagnation. A possible explanation could be that the biofilm in the BAC adapted to the regular interruption of flow (Choi and Morgenroth 2003, Douterelo et al. 2013, Manuel et al. 2009) and therefore, the change of shear conditions did not lead to a significant sloughing off of biofilm at the beginning of the flow period. Continuous detachment of cells has also been identified as the main cause for the high cell concentration in the first flush of other systems with regularly occurring stagnation (Besmer and Hammes 2016). However, for systems where stagnation is a rare event and the biofilm is not adapted to sudden changes in flow conditions, changing shear stress is considered an important cause of biofilm detachment (Paul et al. 2012).

### 4.4 Relevance for hygiene of the reuse water

Water quality is influenced by the location at which water stagnates in the system: biofilter or polyethylene pipe. TCC and turbidity were highest in the water that stagnated in the filter bed. By contrast, organic carbon was lowest in the water that stagnated in the BAC. The water that stagnated in the filter bed had a much longer residence time in the filter bed (480 min instead of typically 30 min) and therefore more of the organic carbon was degraded. Yet, constructing a BAC more than tenfold larger to reach a tenfold longer retention time would only increase the TOC removal from an average of 68% to the observed maximum of 75%. The water quality did not deteriorate in terms of TOC due to stagnation in the BAC.

The microbial communities on biofilters for water treatment play an important role in shaping the microbial community of the effluent water (Pinto et al. 2012). This study focused on two opportunistic pathogens, *L. pneumophila* and *P. aeruginosa*, and on the extent to which the BAC promotes their growth and hence the degree to which detachment of biofilm is detrimental to the hygienic quality of the reuse water. *L. pneumophila* and *P. aeruginosa* concentrations are potentially critical to safe water reuse (Blanky et al. 2017, Coronel-Olivares et al. 2011). However, these pathogens are mentioned very few times in water reuse frameworks (e.g., Spanish Regulation for Water Reuse). Pathogen concentrations in all the samples of the influent and effluent of the BAC are high for any intended reuse involving direct contact with users. The values measured during this one peak event were above or just below regulatory values. Therefore, these pathogens should not be neglected when assessing the safety of the water for reuse. The high concentration in the influent of the BAC shows that these opportunistic pathogens grow before the BAC, in the pipes between the membrane and the BAC. Consequently, if growth after the membrane cannot be prevented, a disinfection step is necessary after the BAC to ensure the hygienic quality of the reuse water. The highest values for both pathogens were measured in the water that stagnated in front of the BAC filter. Further studies are needed to show how stagnation in the pipes between the membrane and the BAC filter influences the growth of these opportunistic pathogens. The numbers do not change for either pathogen because of the BAC treatment, meaning that they grow not in the filter itself but in the pipes and tubes between the membrane and the BAC. *Legionella* has been shown to grow in biofilms with a pH in the range from 5 to 8.5 (EPA 2016). The temperature range in the BAC of 21°C to 23°C is quite low, considering *Legionella* prefer temperatures between 35°C and 42°C (Rhoads et al. 2016). In contrast, *P. aeruginosa* can grow optimally at temperatures between 10°C and 42°C and at low nutrient concentrations (Bédard et al. 2016); hence, this pathogen is well adapted to growth in the BAC filter. Nevertheless, both the finding that the concentrations in the BAC effluent are not higher in the effluent than in the influent and that the peak in TCC does not correlate with increased pathogen numbers are strong indicators that the biofilter is not a hotspot for the growth of these opportunistic pathogens. Therefore, in our case, the BAC is not of concern for the growth of these opportunistic pathogens.

After stagnation in the BAC, a deterioration in water quality was observed in terms of cell concentration and turbidity. But it is important to note that the increased cell concentration is not related to the pathogens investigated in this study or to an increase in effluent TOC concentrations. Hence, stagnation does not lead to a deterioration of the parameters relevant to ensuring the hygiene of reuse water. Nevertheless, increased turbidity can lead to decreased disinfection performance, for example with UV disinfection (Farrell et al. 2018). Guidelines from the World Health Organization suggest that turbidity values above 0.2 NTU can negatively influence disinfection performance (World Health Organization 2017). Results such as ours that show a clear pattern of turbidity between 0.2 and 1 FNU should therefore be taken into consideration when a disinfection step is designed to ensure sufficient disinfection at all times and to prevent any negative impacts of increased turbidity on performance.

## 5 Conclusion

This study presents four main conclusions:

- Highly variable flow into household-scale greywater treatment systems leads to periods with stagnant water. After stagnation, increased turbidity and cell concentrations are measured in the effluent of the BAC.
- Stagnation does not lead to an increase in opportunistic pathogens in the effluent, showing that the BAC is not a hotspot for the growth of opportunistic pathogens *L. pneumophila* and *P. aeruginosa*. Therefore, no additional measures are necessary to exclude the first water after stagnation from reuse.
- After the BAC, turbidity correlates strongly with cell concentrations. Therefore, turbidity can be used as a proxy for increased cell concentrations, and unexpected fluctuations can be an indication of process failure.
- The extent and fluctuations of increased cell concentrations and turbidity are consistent with a model that assumes that the rate of continuous detachment in the BAC is higher during periods with flow than during stagnation.

## Declaration of Competing Interest

The authors declare that they have no known competing financial interests or personal relationships that could have appeared to influence the work reported in this paper.

## Appendix Supplementary materials

Image, application 1
