# Supplementary material for: Stagnation leads to short-term fluctuations in the effluent water quality of biofilters: A problem for greywater reuse?
Source: Water Res X. 2021 Sep 15;13:100120. doi: 10.1016/j.wroa.2021.100120 (PMC8495163; doi:10.1016/j.wroa.2021.100120)
Supplement: Supplementary file 1 [file mmc1.docx]

Supporting information for:

Stagnation leads to short-term fluctuations in the effluent water quality of biofilters: A problem for greywater reuse?

*Angelika Hess^a,b^, Chiara Baum^a^, Konstanze Schiessl^c^, Michael Besmer^c^, Frederik Hammes^a^, and Eberhard Morgenroth^a,b,*^*

*^a^: Eawag: Swiss Federal Institute of Aquatic Science and Technology, 8600, Dübendorf, Switzerland*

*^b^: ETH Zürich, Institute of Environmental Engineering, 8093, Zürich, Switzerland*

*^c^: onCyt Microbiology AG, 8038 Zürich, Switzerland*

**Corresponding Author. Email address: Eberhard.Morgenroth@eawag.ch*

First submitted to Water Research X on February 14, 2021

Revised version submitted on July 18, 2021

# Material and Methods

## Turbidity

##
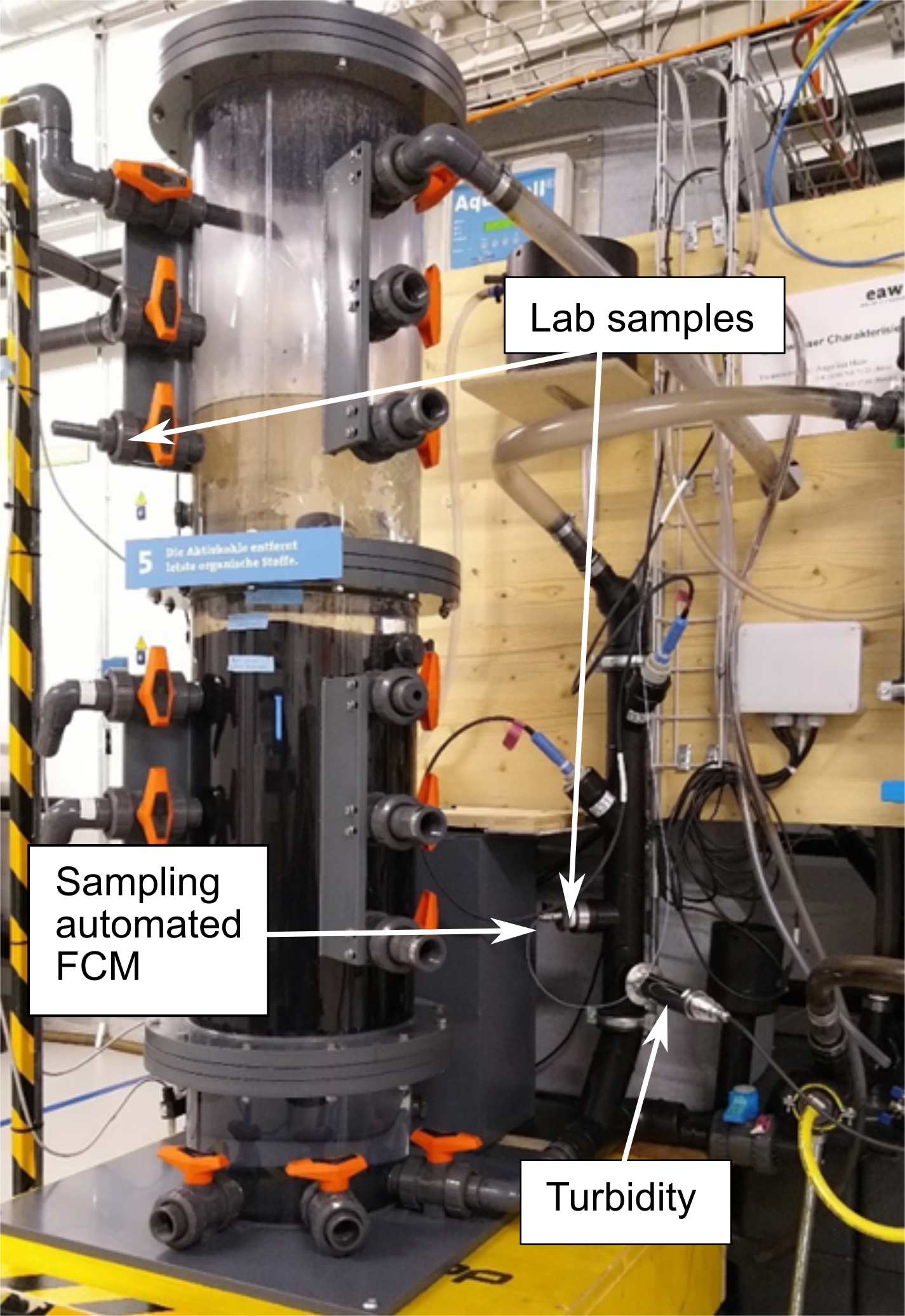


Figure S1: Photo of BAC filter with turbidity sensor and sampling locations.

## Nutrients

##
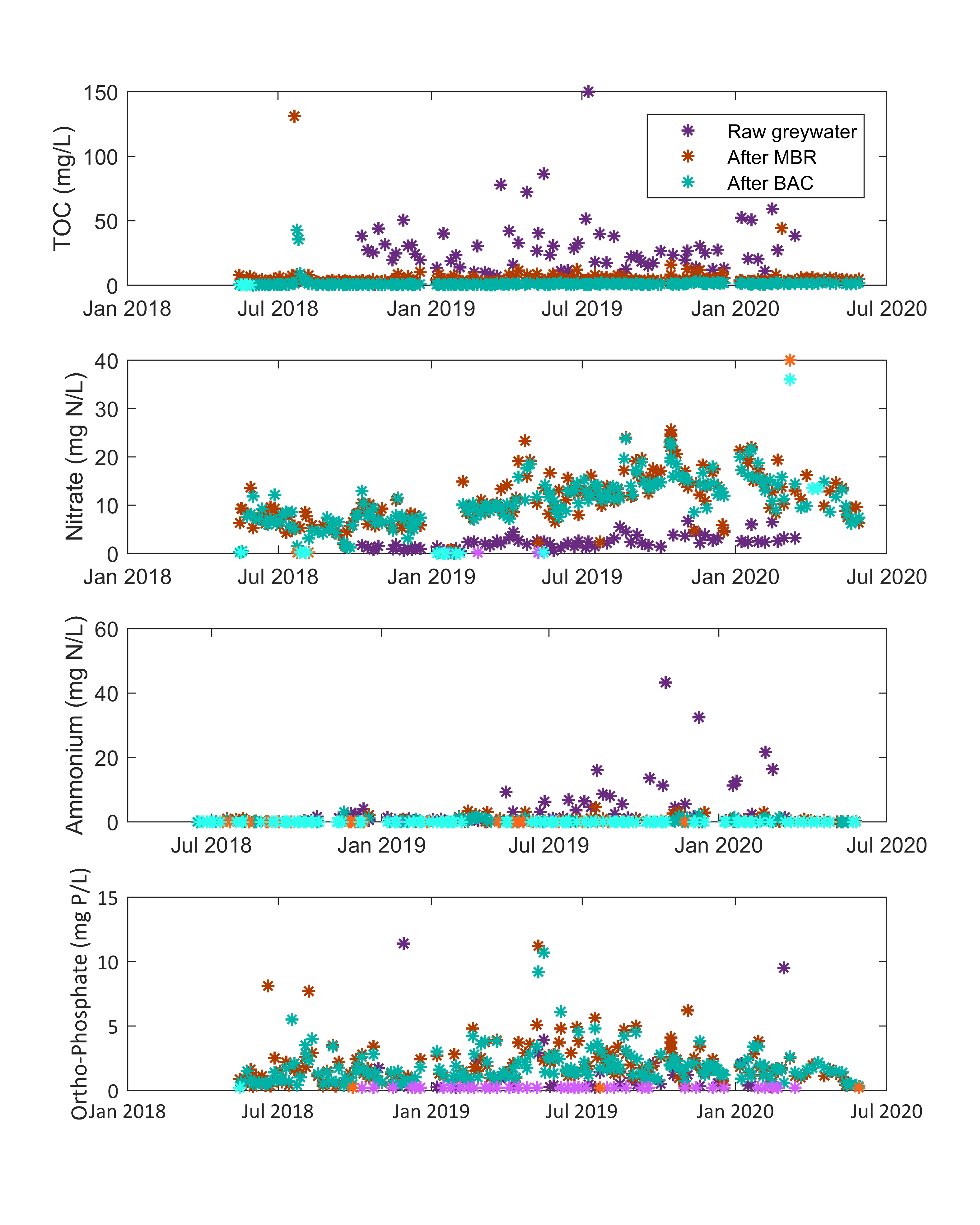


Figure S2: Nutrients measured for the greywater treatment system over time. The light colors indicate values above or below the limit of quantification.

##

## Automated flow cytometry, Outlier removal

Outliers were removed by visually checking the density plots for ‘abnormal’ density plots, which were removed from the dataset.

For orientation on the visual inspection, a normal density plot looks like shown in Figure 2.

Figure S3: Example of a density plot that looks normal.

One type of typical outliers found is a class of samples where only around 20 µl were measured instead of 99 µl, i.e. the FCM was not able to measure successfully and automatically stopped the measurement (e.g. because of a clog). An example of such a density plot is shown in Figure 3.

Figure S4: Example of a density plot that was considered as an outlier.

Another type of outlier are plots that show cells with low fluorescence values, possibly caused by poor staining, as for example seen in Figure 4.

Figure S5: Example of density plot that was considered as an outlier.

Both of these types of outliers were removed.

Statistics on these:

- N (all data points) =3 995
- N (outliers with volume < 80 µl) = 68
- N (other outliers) = 72, i.e. a total of 140 outliers
  1. Comparison conventional FCM and online FCM


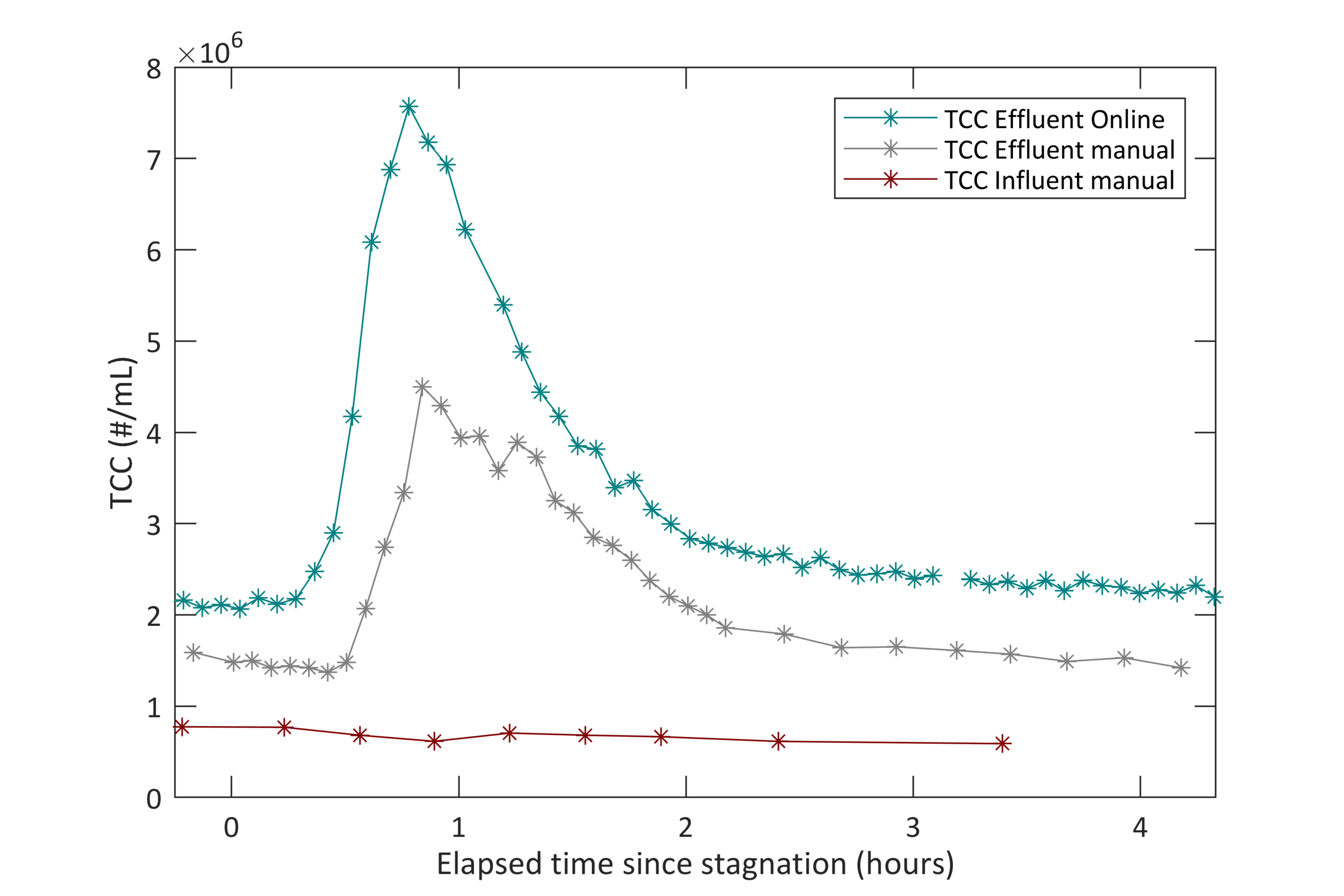


Figure S6: TCC measured with conventional FCM for the influent and the effluent of the BAC. And TCC measured with online FCM for the effluent of the BAC.

The differences between the TCC measured with online FCM and manual FCM can be explained with: i) the samples for manual measurements were fixed with glutaraldehyde and formaldehyde and ii) different instrument had to be used for online FCM and conventional FCM.

## Modelling

- - 1. Flow through the filter

Flow was calculated based on pressure changes measured with a pressure sensor (Cerabar T PMC131) in the water head on top of the BAC filter. The flow is pumped in batches of 6.1 L from the MBR to the BAC, leading to continuous change in pressure on top of the filter bed.

$$Flow(t)=\frac{p(t)-p(t-1)}{t-(t-1)}\cdot0.74$$

, with 0.74 (L/mbar) the factor to convert the measured pressure to volume.

For$p(t)-p(t-1)<0$:

$$Q_{out}(t)=|Flow(t)|$$

For$p(t)-p(t-1)\geq0$:

$$Q_{out}(t)=Q_{out}(t_{p})$$

$$Q_{in}(t)=Flow(t)+Q_{out}(t)$$

, with $t_{p}$ the time when the measured pressure was closest to $p(t)$ during the last week. For these we assume that the hydraulic resistance over the filter bed for a given pressure does not change over a period of one week.

- - 1. Model of the filter

The top of the filter is modelled as a completely mixed tank with varying volume:

$V_{top}(t)=V_{top}(t-1)+(p(t)-p(t-1))\cdot\frac{D_{Filter}^{2}}{4}\cdot\pi$,

$V_{top}$ = volume of the water head above the filter bed,
$p$ = pressure measured on top of the filter in meter water head, and
$D_{Filter}$ = filter diameter

This leads to the following concentrations in the water head above the filter bed:

$C_{top}(t)=C_{top}(t-1)$ , if $Q_{in}=0$

$C_{top}(t)=\frac{C_{top}(t-1)\cdot V_{top}(t-1)+C_{in}\cdot Q_{in}(t-1)\cdot\Delta t-C_{top}(t-1)\cdot Q_{out}(t-1)\cdot\Delta t}{V_{top}(t)}$ , if $Q_{in}>0$

$C_{top}$ = Concentration in the water head above the filter bed

The filter bed is modelled as a plug flow reactor (PFR) modelled as a cascade of completely mixed stirred reactors (CSTRs):

For the first compartment (n = 1) this results in:

$C_{Filter}(t,1)=C_{Filter}(t-1,1)$ , if $Q_{out}=0$

$C_{Filter}(t,1)=C_{Filter}(t-1,1)+\frac{Q_{out}(t)\cdot\Delta t}{V_{Filter}/n_{Filter}}\cdot(C_{top}(t-1)-C_{Filter}(t-1,1))$ , if $Q_{out}>0$

And for the following compartments (n = 2 to n= 8):

$C_{Filter}(t,n)=C_{Filter}(t-1,n)$ , if $Q_{out}=0$

$C_{Filter}(t,n)=C_{Filter}(t-1,n)+\frac{Q_{out}(t)\cdot\Delta t}{V_{Filter}/n_{Filter}}\cdot(C_{Filter}(t-1,n-1)-C_{Filter}(t-1,n))$ , if $Q_{out}>0$

$C_{Filter}$ = Concentration in the filter bed in the n^th^ compartment
$V_{Filter}$ = Volume of the filter bed, corrected with the porosity
$n_{Filter}$ = number of compartments that are modelled as CSTRs. Here we chose n = 8

The water volume at below the filter bed was modelled as a CSTR:

$C_{bottom}(t)=C_{bottom}(t-1)$ , if


, if $Q_{out}>0$

$C_{bottom}$ = Concentration in the water volume below the filter bed
$V_{bottom}$ = Volume of the water volume below the filter bed
The upflow pipe between the bottom of the filter and the measurement and sampling location was modelled as a PFR, modelled as a series of CSTRs:

For the first compartment (n = 1) this results in:

$C_{pipe}(t,1)=C_{pipe}(t-1,1)$ , if $Q_{out}=0$

$C_{pipe}(t,1)=C_{pipe}(t-1,1)+\frac{Q_{out}(t)\cdot\Delta t}{V_{pipe}/n_{pipe}}\cdot(C_{bottom}(t-1)-C_{pipe}(t-1,1))$ , if $Q_{out}>0$

And for the following compartments (n = 2 to n= 10):

$C_{pipe}(t,n)=C_{pipe}(t-1,n)$ , if $Q_{out}=0$


$C_{pipe}(t,n)=C_{pipe}(t-1,n)+\frac{Q_{out}(t)\cdot\Delta t}{V_{pipe}/n_{pipe}}\cdot(C_{pipe}(t-1,n-1)-C_{pipe}(t-1,n))$ , if $Q_{out}>0$

$C_{Filter}$ = Concentration in the filter bed in the n^th^ compartment
$V_{Filter}$ = Volume of the filter bed, corrected with the porosity
$n_{Filter}$ = number of compartments that are modelled as CSTRs. Here we chose n = 8

The hydraulic model of the filter was verified with a tracer experiment adding 2.54 g NaCl (Merck). In the effluent of the filter, conductivity was measured at a 10 s interval and 7 samples were analysed in the lab for sodium and chloride. Data from the hydraulic model and the tracer data can be found in the Supporting information.

- - 1. Tracer test


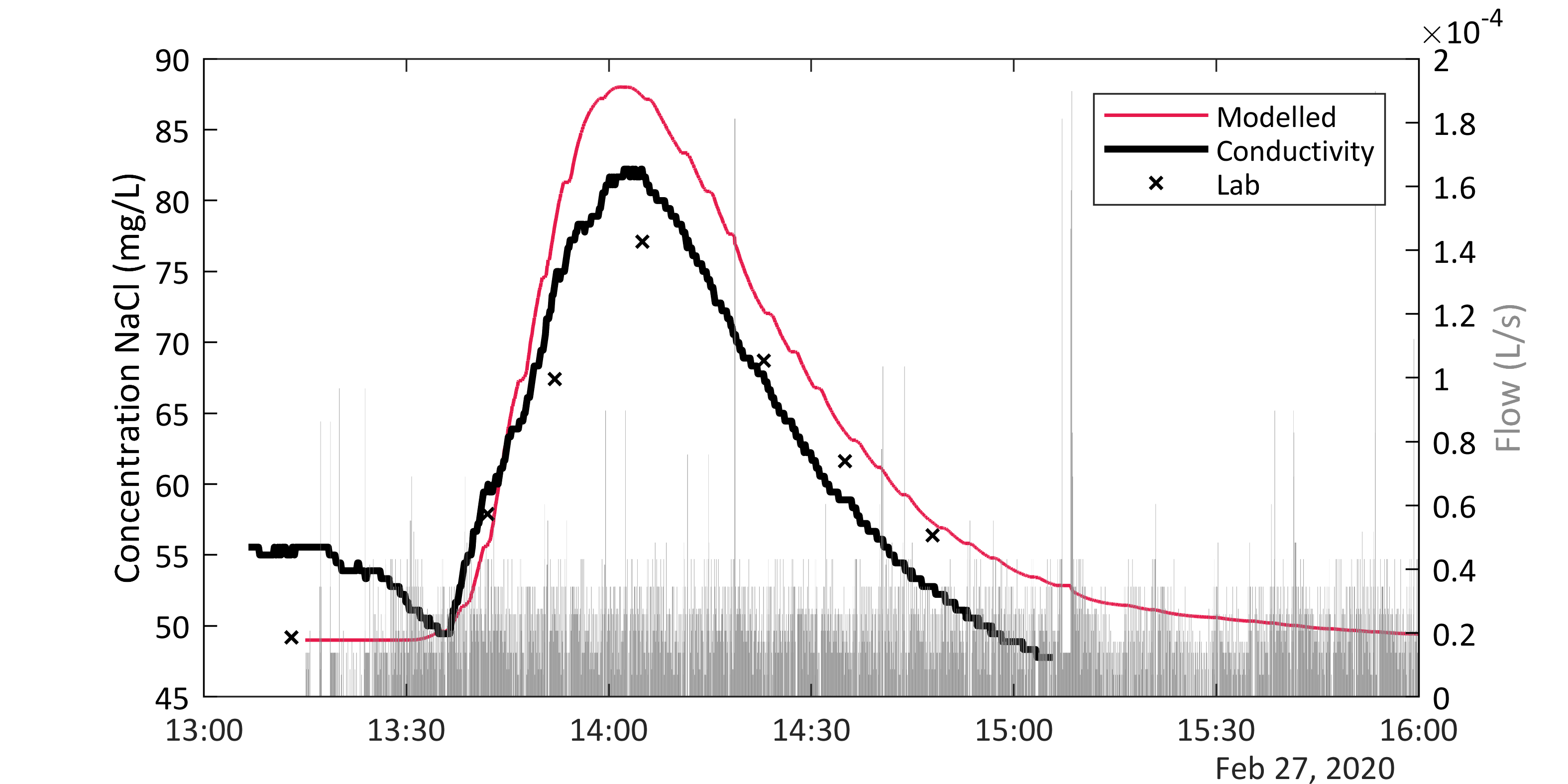


Figure S7: Tracer test to verify the hydraulic model of the BAC.

The hydraulic model was tested with a tracer test using NaCl as a tracer, added to the water volume on top of the filter bed. The effluent NaCl concentration was estimated based on conductivity measurements and with additional lab samples.

- - 1. ATP measurements

ATP was measured as described in Hess et al. (2020). Samples from the height of 7 cm, 22 cm, and 37 cm were analysed for ATP on the GAC.

The ATP concentrations were 4.76·10^-6^ gATP/gGAC, 1.76·10^-6^ gATP/gGAC, and 7.28·10^-7^ gATP/gGAC for 7, 22, and 37 cm, respectively. An exponential function was fitted to the measured data and with this function, the ATP concentration for the different compartments was estimated.

# Results

## SEM microscopy

For microscopy, 500 mL of BAC effluent was filtered on a 0.45 µm filter and analysed.


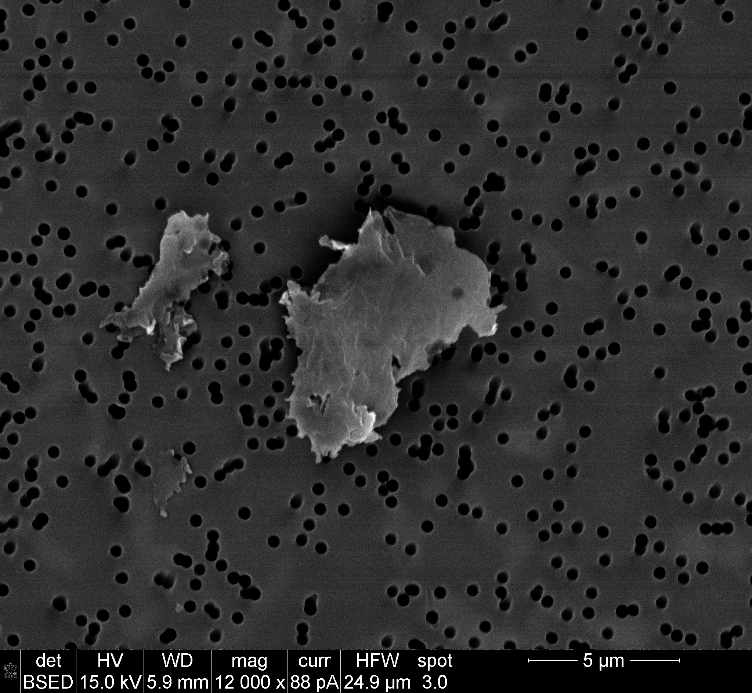


Figure S8: Picture of two flat Si particles.


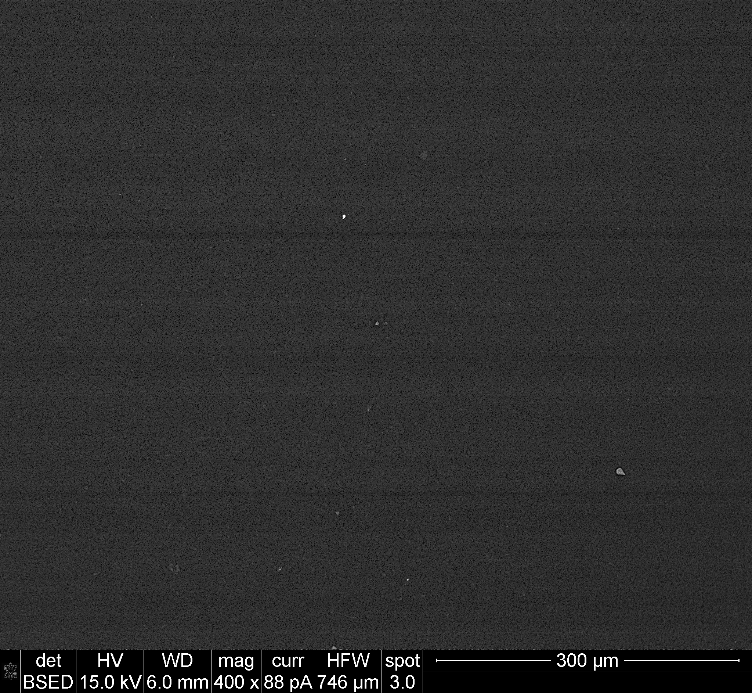


Figure S9: Overview of the sample. The white dots show chalk particles.


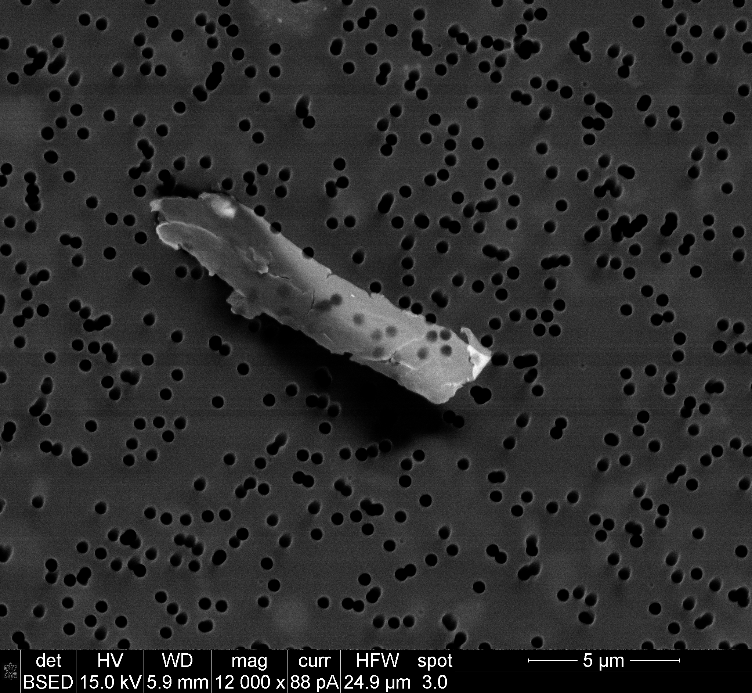


Figure S10: Carbon fibre detected in the effluent of the BAC.

## TCC-Turbidity relation with pure culture


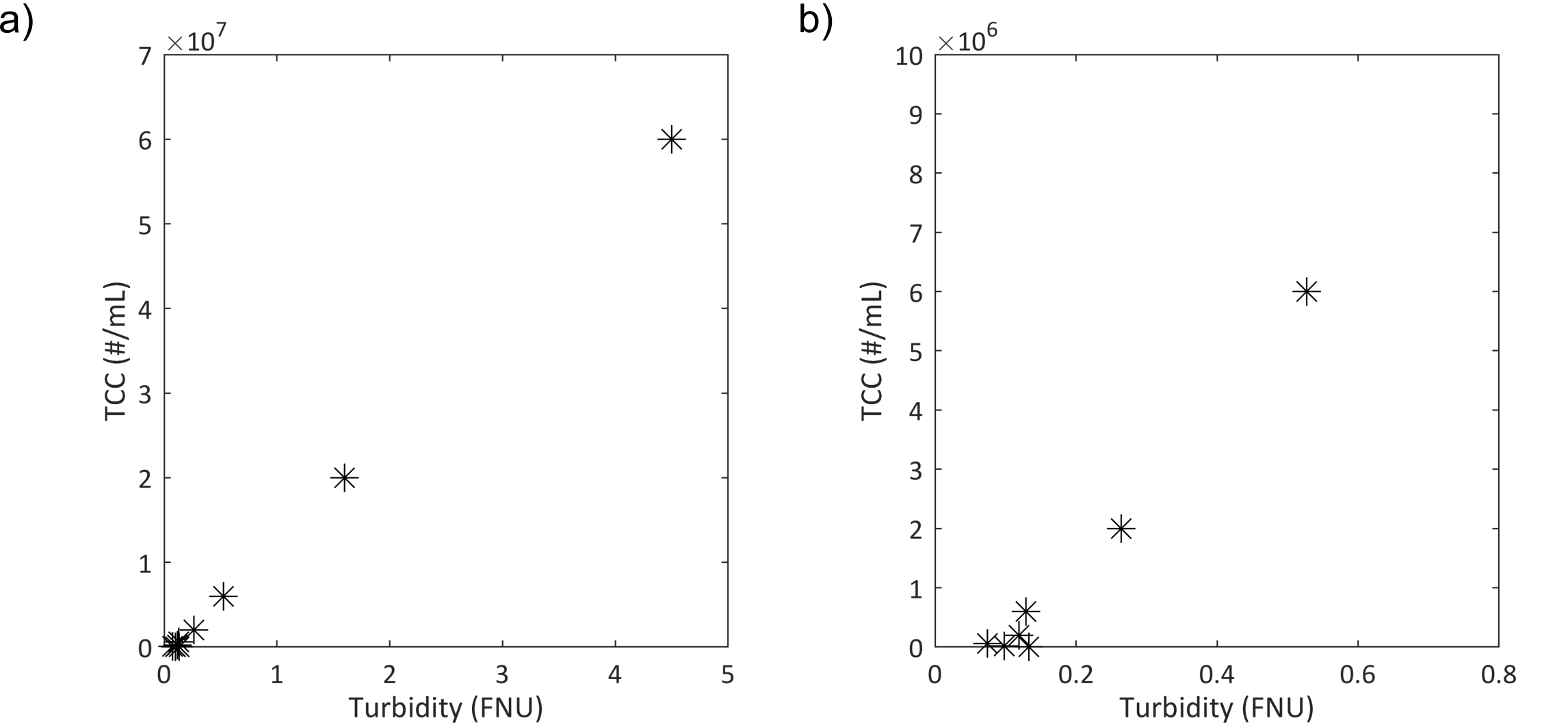


Figure S11: Turbidity and cell concentration measured for a dilution series with a bacteria pure culture. A) shows all the measured data points and b) a magnification for the lower turbidity range.

## Continuous detachment of cells from the BAC


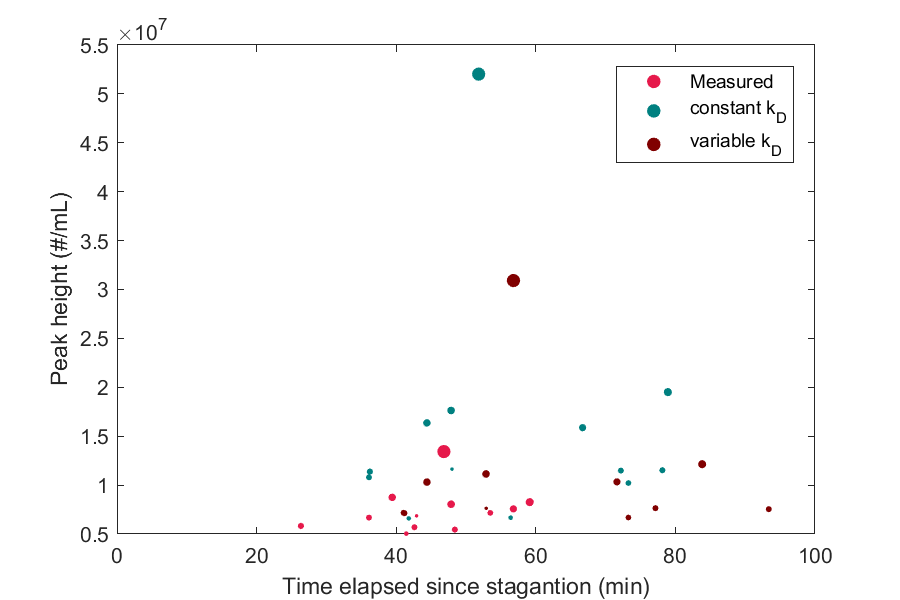


Figure S12: Comparison of measured and modelled peak height for a given time elapsed after stagnation. The larger the circle, the longer the stagnation time was.

## Relevance for hygiene of the reuse water?

For the peak where different parameters were measured, a correlation matrix was performed. The correlation matrix shows if the different parameters measured in the effluent of the BAC show significant correlation or not.

Figure S13: Correlation matrix for the different variables measured during a TCC peak. The numbers show the R2, the red colour indicates statistically significant correlations (i.e. p<0.5).

# References

Hess, A., Bettex, C. and Morgenroth, E. (2020) Influence of intermittent flow on removal of organics in a biological activated carbon filter (BAC) used as post-treatment for greywater. Water Research X 9, 100078.
